# Evaluation of Biological Properties of Electron Beam Melted Ti6Al4V Implant with Biomimetic Coating In Vitro and In Vivo

**DOI:** 10.1371/journal.pone.0052049

**Published:** 2012-12-18

**Authors:** Xiang Li, Ya-Fei Feng, Cheng-Tao Wang, Guo-Chen Li, Wei Lei, Zhi-Yong Zhang, Lin Wang

**Affiliations:** 1 School of Mechanical Engineering, Shanghai Jiao Tong University, State Key Laboratory of Mechanical System and Vibration, Shanghai, China; 2 Department of Orthopaedics, Xijing Hospital, The Fourth Military Medical University, Xi’an, China; 3 Department of Orthopaedics, Tangdu Hospital, The Fourth Military Medical University, Xi’an China; 4 Department of Plastic and Reconstructive Surgery, Shanghai 9th People’s Hospital, Shanghai Key Laboratory of Tissue Engineering, School of Medicine, Shanghai Jiao Tong University, Shanghai, China; University of Akron, United States of America

## Abstract

**Background:**

High strength porous titanium implants are widely used for the reconstruction of craniofacial defects because of their similar mechanical properties to those of bone. The recent introduction of electron beam melting (EBM) technique allows a direct digitally enabled fabrication of patient specific porous titanium implants, whereas both their in vitro and in vivo biological performance need further investigation.

**Methods:**

In the present study, we fabricated porous Ti6Al4V implants with controlled porous structure by EBM process, analyzed their mechanical properties, and conducted the surface modification with biomimetic approach. The bioactivities of EBM porous titanium in vitro and in vivo were evaluated between implants with and without biomimetic apatite coating.

**Results:**

The physical property of the porous implants, containing the compressive strength being 163 - 286 MPa and the Young’s modulus being 14.5–38.5 GPa, is similar to cortical bone. The in vitro culture of osteoblasts on the porous Ti6Al4V implants has shown a favorable circumstance for cell attachment and proliferation as well as cell morphology and spreading, which were comparable with the implants coating with bone-like apatite. In vivo, histological analysis has obtained a rapid ingrowth of bone tissue from calvarial margins toward the center of bone defect in 12 weeks. We observed similar increasing rate of bone ingrowth and percentage of bone formation within coated and uncoated implants, all of which achieved a successful bridging of the defect in 12 weeks after the implantation.

**Conclusions:**

This study demonstrated that the EBM porous Ti6Al4V implant not only reduced the stress-shielding but also exerted appropriate osteoconductive properties, as well as the apatite coated group. The results opened up the possibility of using purely porous titanium alloy scaffolds to reconstruct specific bone defects in the maxillofacial and orthopedic fields.

## Introduction

Titanium and titanium alloys have been widely used in orthopedic and dental implants due to low density, excellent mechanical properties, favorable biocompatibility, and good corrosion resistance. However, clinical practices and studies have shown that the mechanical mismatch between metallic implant and natural bone may lead to stress-shielding, and thus cause bone resorption and eventually the failure of metallic implant fixation [1]. Porous metallic structure can be utilized to overcome this drawback, which not only reduced the mechanical mismatch but also achieved stable long-term fixation by promoting full bone ingrowth [2], [3]. Many techniques have been investigated to produce porous metallic structure, including powder sintering approach, space holder method, combustion synthesis, plasma spraying, and polymeric sponge replication [3]–[6]. However, these conventional techniques have very limited control of the internal pore architecture and the external shape of the porous titanium implants, which hinder further application of porous titanium.

**Figure 1:**
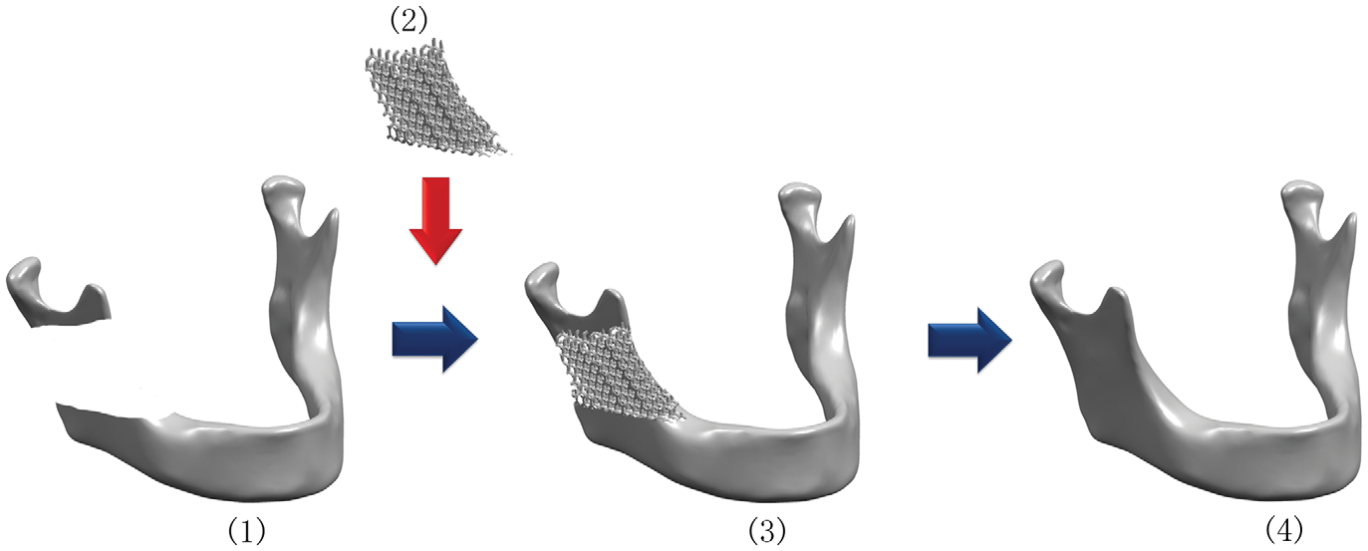
The flow diagram showed the design of electron beam melting (EBM) porous Ti6Al4V implant with CAD for repair of mandibular bone defect: (**1**) acquisition of the CT data of the patients; (**2**) design with CAD and fabrication of custom EBM porous titanium implant; (**3**) implantation of the patient specific porous implant; (**4**) reconstruction of the bone defect.

Rapid prototyping (RP), generally known as solid freeform fabrication (SFF), is a type of technologies that can automatically construct physical models from computer-aided design (CAD) data. The applications of state-of-the-art RP techniques for fabricating polymeric tissue engineering (TE) scaffolds were reviewed by Leong and Hutmacher [7], [8], with detailed illustration to show the superiority of RP techniques over the conventional fabrication methods. Direct fabrication of metallic components for biomedical application with RP approaches, such as three-dimensional fiber deposition [9], laser-engineered net shaping [10], direct laser forming [11] and so on, has been shown to become a feasible and promising manufacturing technology in producing porous Ti6Al4V scaffolds with interconnected porous networks. Recently, selective electron beam melting (EBM) approach as a metal rapid prototyping process has been studied for fabricating patient specific porous orthopedic implants [12]. The EBM porous titanium implant not only avoided the stress-shielding effects in vivo for similar mechanical properties with native bone [13], but also matched irregular defect at specific site such as skull, maxillofacial and bone joint region [Fig. 1]. Heinl et al. has reported that cellular Ti6Al4V structures with interconnected macro porosity fabricated by EBM might have favorable long-term stability and were suitable for orthopedic applications [14].

In addition to the internal structure and external shape, the implant surface physicochemical properties are also critical for bone-implant integration. As solid titanium and alloys are generally encapsulated by fibrous tissue after implantation for their bioinert nature [15], [16], many surface modification methods, including plasma spraying [17], sol-gel [18], electrophoretic deposition [19], sputter deposition [20] and micro-arc oxidation [21], were applied to improve the bone-implant integration of this kind of implants. The biomimetic approach, which via soaking implants in simulated body fluids (SBF) at a physiological temperature and pH, has been shown to be a superior option due to its low operation temperature and homogenously bone-like carbonated apatite deposition [22]–[24]. Barrere et al. [25] reported that the biomimetic coating enhanced the implant-bone integration and were beneficial for the long-term fixation of metal prostheses in the load-bearing applications. However, few studies have analyzed the biological performance of EBM porous titanium implants with biomimetic coating.

The aim of this study was to fabricate Ti6Al4V implants with well controlled porous structure using EBM process, and to evaluate the bioactivity of this porous implant with or without biomimetic apatite coating in vivo and in vitro. The implant physical properties were examined by scanning electron microscopy, micro computer tomography, and digital microscope. The implant surfaces were modified by biomimetic approach and characterized by energy-dispersive X-ray analysis, X-ray diffraction and Fourier transform infrared spectroscopy. In vitro, experiments were performed to assess cell attachment, viability, cell proliferation and differentiation. In vivo, samples were implanted into 12 mm bone defects in calvaria of rabbits, and bone ingrowth and bone implant bonding was evaluated by histology and histomorphometry.

## Materials and Methods

### Preparation of Porous Ti6Al4V Implant

Porous Ti6Al4V implant was fabricated using electron beam melting process as previously described [12]. Briefly, the external shape and internal porous structures were designed with commercial CAD software (Unigraphics NX, EDS). The CAD data of the structures was converted into STL data which then imported into Materialise’s Magics software, and converted into input file for EBM. The samples were produced on an Arcam’s EBM machine (EBM S12, Arcam AB, Sweden).

**Figure 2:**
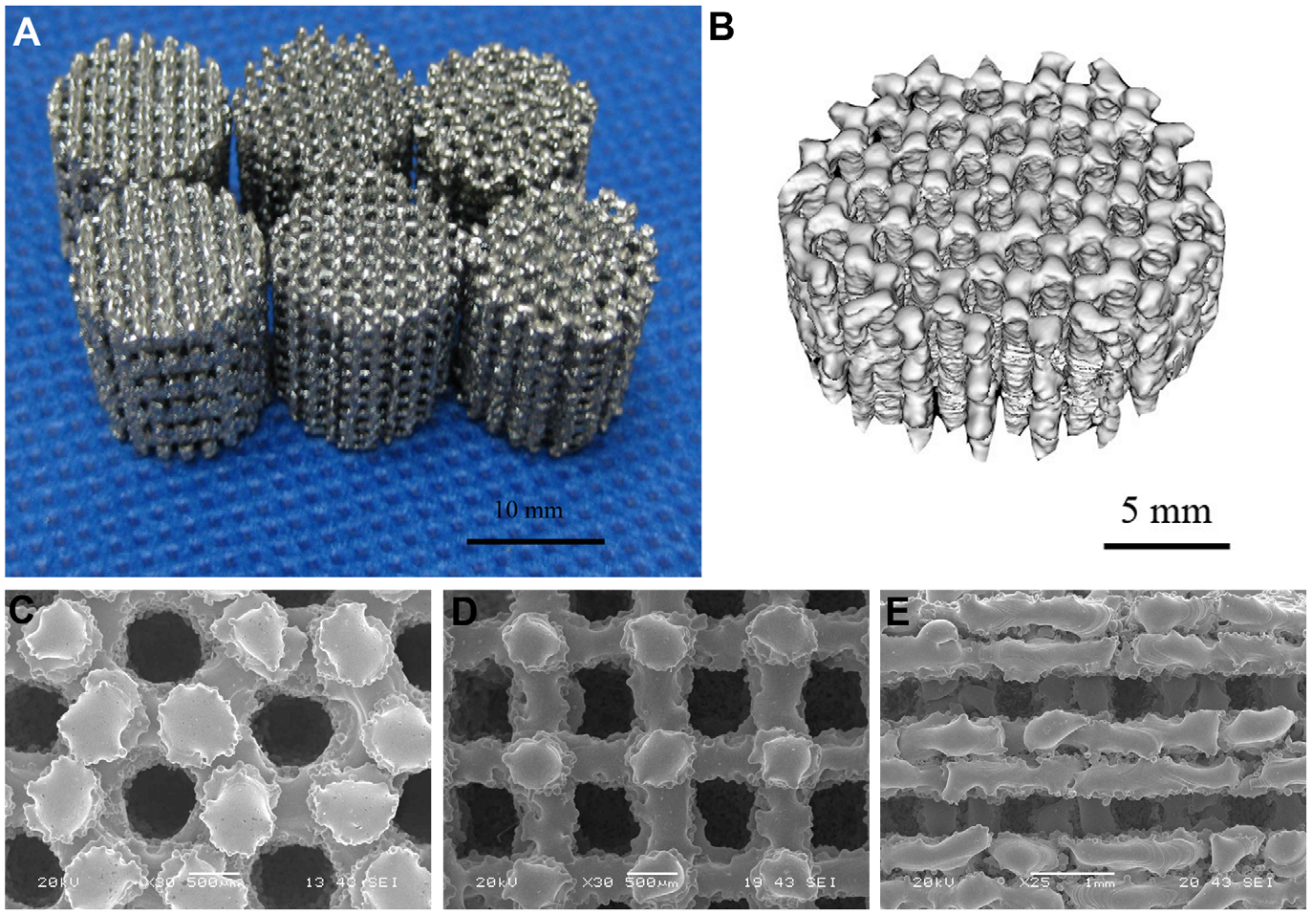
Characterization of porous Ti6Al4V samples. (**A**) Porous Ti6Al4V implants fabricated by electron beam melting process. (**B**) Reconstructed 3D micro-CT image of the porous implant with honey-like structure. SEM images of porous Ti6Al4V samples with (**C**) honeycomb-like structure, (**D**) orthogonal structure, (**E**) layer structure.

**Figure 3:**
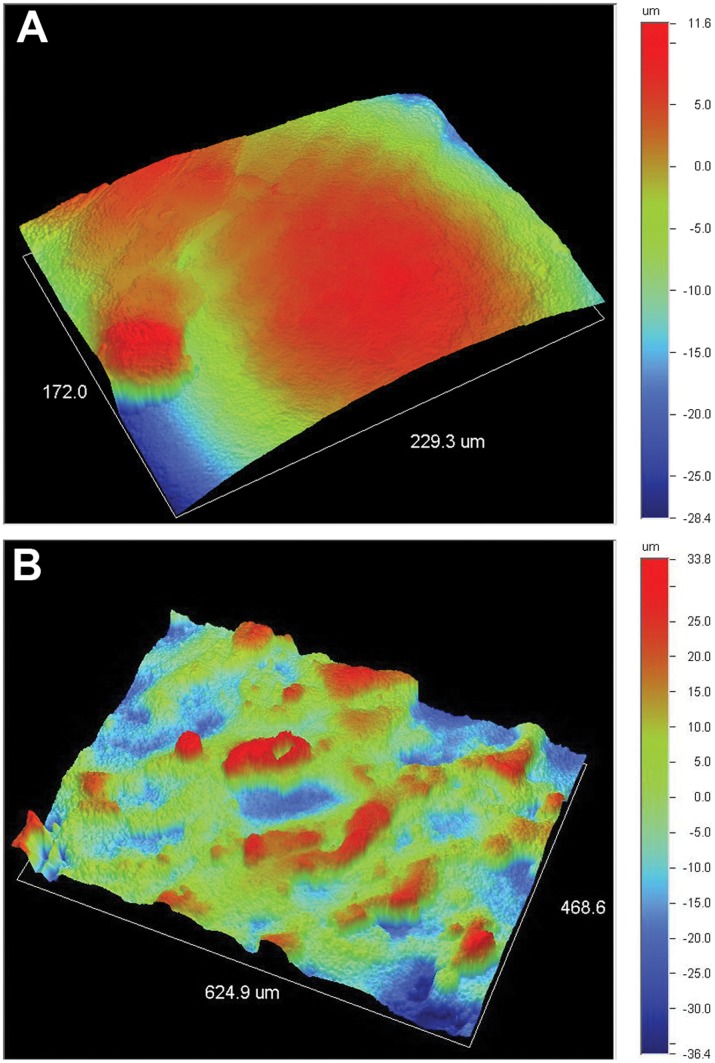
Digital topographic images of the sample surface. The images exhibited a rough anisotropic surface with the roughness *R_a_* values of the (**A**) top and (**B**) lateral surfaces being in the range of 5–10 and 15–21 µm.

**Table 1:** Geometric characteristics and mechanical properties of porous titanium samples.

	Honey-like structure	Orthogonal structure	Layering structure	Cortical bone
Porosity (%)	51.4±2.4	61.2±3.7	55.3±2.6	
Pore size (µm)	∼600	∼600	∼500	
Strut size (µm)	∼650	∼500	∼900	
E (GPa)	25.9±2.8	14.5±3.1	38.5±6.7	10–30
σ_0.2_ (MPa)	185.4±15.9	138.2±8.6	194.4±12.1	∼138
σ_max_ (MPa)	286.6±35.5	163.8±11.3	235.6±18.3	∼193

E, the elastic modulus.

σ_0.2_, the compressive yield strength.

σ_max_, the compressive maximum strength.

### Characterization of Porous Ti6Al4V Implant

The porous structures of the EBM produced samples were observed using a scanning electron microscope (SEM, JSM-6460, JEOL, Japan) equipped with an energy dispersive X-ray analyzer (EDX, Oxford, UK). The structural analysis using micro computer tomography (µCT, Locus SP, GE, USA) was conducted prior to the chemical treatments to determine the mean pore size and the porosity of the implants. The resolution was 20 µm. The three-dimensional (3D) surface topography of the samples was examined by optical profiler (OP, Wyko NT9300, Veeco, USA). Five samples were measured from each of the two different surfaces (top&bottom and lateral) to obtain an average roughness value *R_a_*. The porosity of EBM produced samples was evaluated from the weight and the apparent volume of the specimen (*n* = 5). Compression tests were conducted to determine the mechanical properties of the EBM Ti6Al4V implants (*n* = 5) using a material testing system (MTS 810) with a 10 kN load cell at room temperature. The cross-head speed was set at 0.5 mm/min. The elastic modulus E was calculated from the slope of the compressive stress–strain curve in the linear elastic region. The compressive yield strength σ_0.2_ was determined from the stress–strain curve according to the 0.2% offset method, while the maximum strength σ_max_ was calculated by dividing the highest load by the initial cross-sectional area.

**Figure 4:**
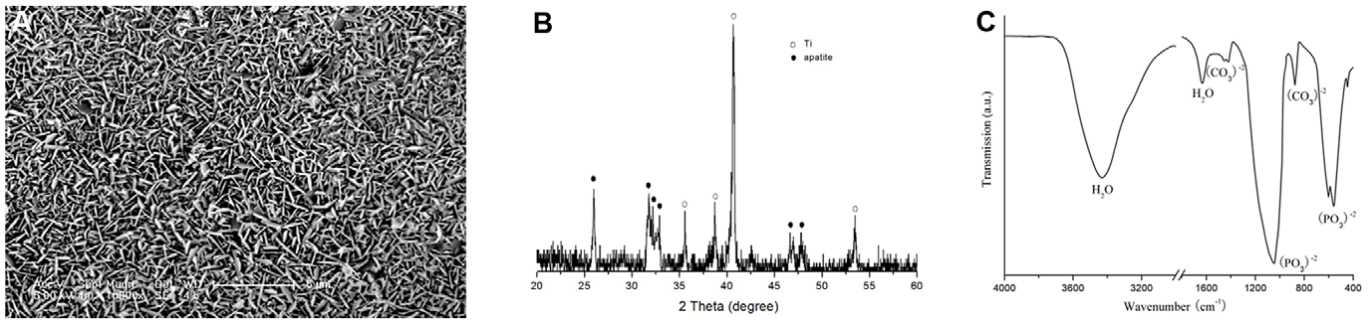
Characterization of porous titanium with biomimetic coating. (**A**) SEM image of the precipitate on sample surfaces soaked in SBF for 14 days. (**B**). TF-XRD pattern of the sample surface after alkali-heat treatment and subsequently immersion in SBF for 14 days. (**C**) FTIR spectra of chemically pretreated Ti6Al4V samples soaked in SBF for 14 days.

**Figure 5:**
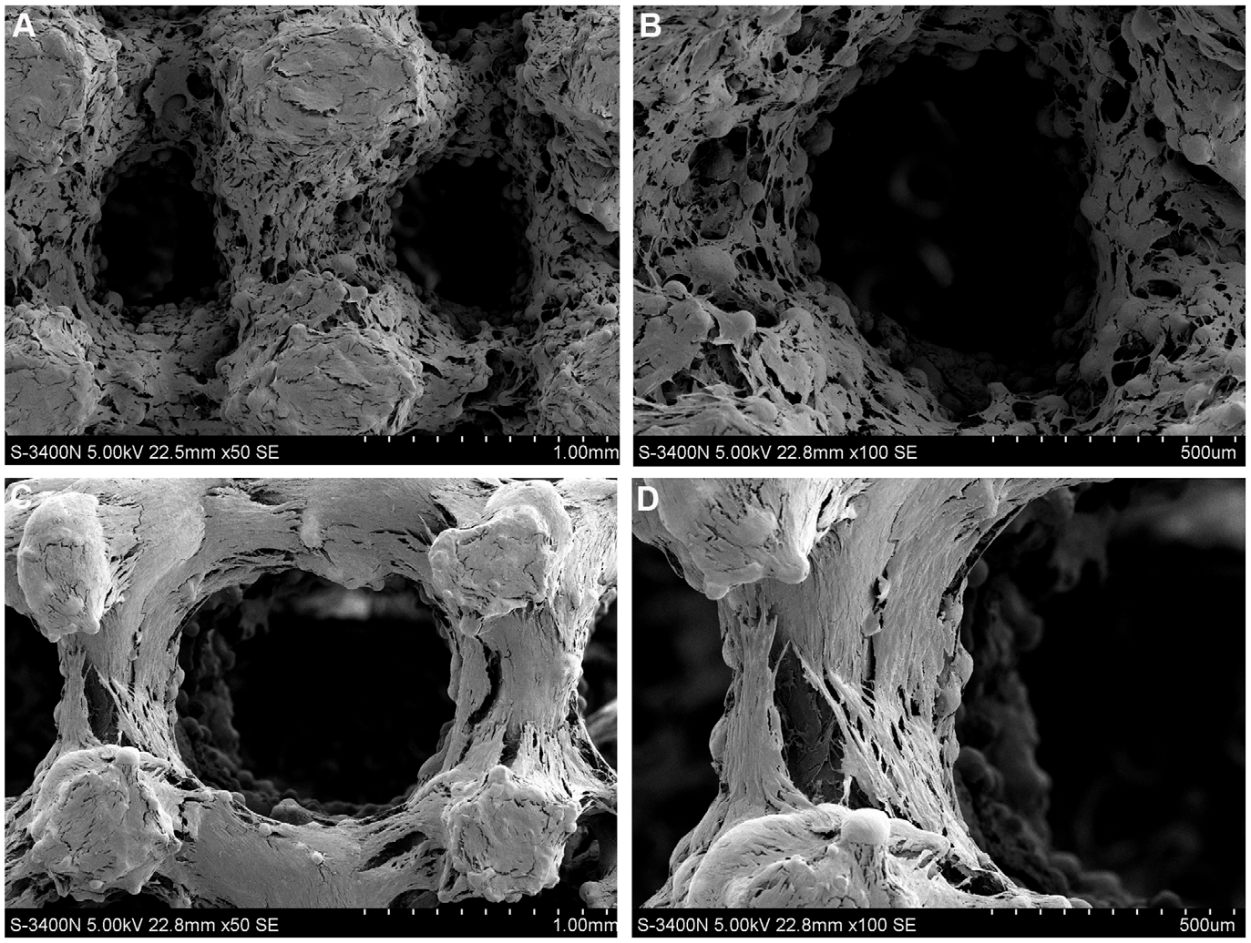
SEM morphologies of cells on porous titanium samples after 14 days of culture. A great number of osteoblasts attached to the (**A–B**) pure porous titanium scaffolds and (**C–D**) porous titanium scaffolds with biomimetic coating, and presented an elongated morphology with cytoplasmic extensions on scaffolds. There were no obvious differences in cell adhesion and morphology between the uncoated and coated samples.

### Biomimetic Coating

Samples for surface modification were machined to cylinder-shape with diameter in 11.6 mm and height in 5 mm. Before chemical treatment, samples were washed with isopropanol and distilled water in an ultrasonic cleaner, and then dried in air. Alkali treatment was performed by soaking these samples in 10 *M* NaOH aqueous solution at 60°C for 24h, then washed gently with distilled water. The alkali-treated samples were heated to 600°C (at a rate of 5°C min^−1^) in an electric furnace, kept at 600°C for 1h, and then allowed to cool to room temperature in the furnace [23]. The alkali-heat treated samples were immersed in SBF solution under static conditions at 37°C for 14 days. The solution was changed every 2 days to maintain the solution concentrations. The SBF solution was prepared according to Kokubo et al’s method [26]. Briefly, reagent grade chemicals of NaC1, NaHCO_3_, KCl, K_2_HOP_3_·3H_2_O, MgC1_2_·6H_2_O, CaC1_2_, and Na_2_SO_4_ were dissolved into distilled water, and buffered at pH 7.40 with hydrochloric acid and tris-hydroxymethyl aminomethane at 36.5±1.5°C.

**Figure 6:**
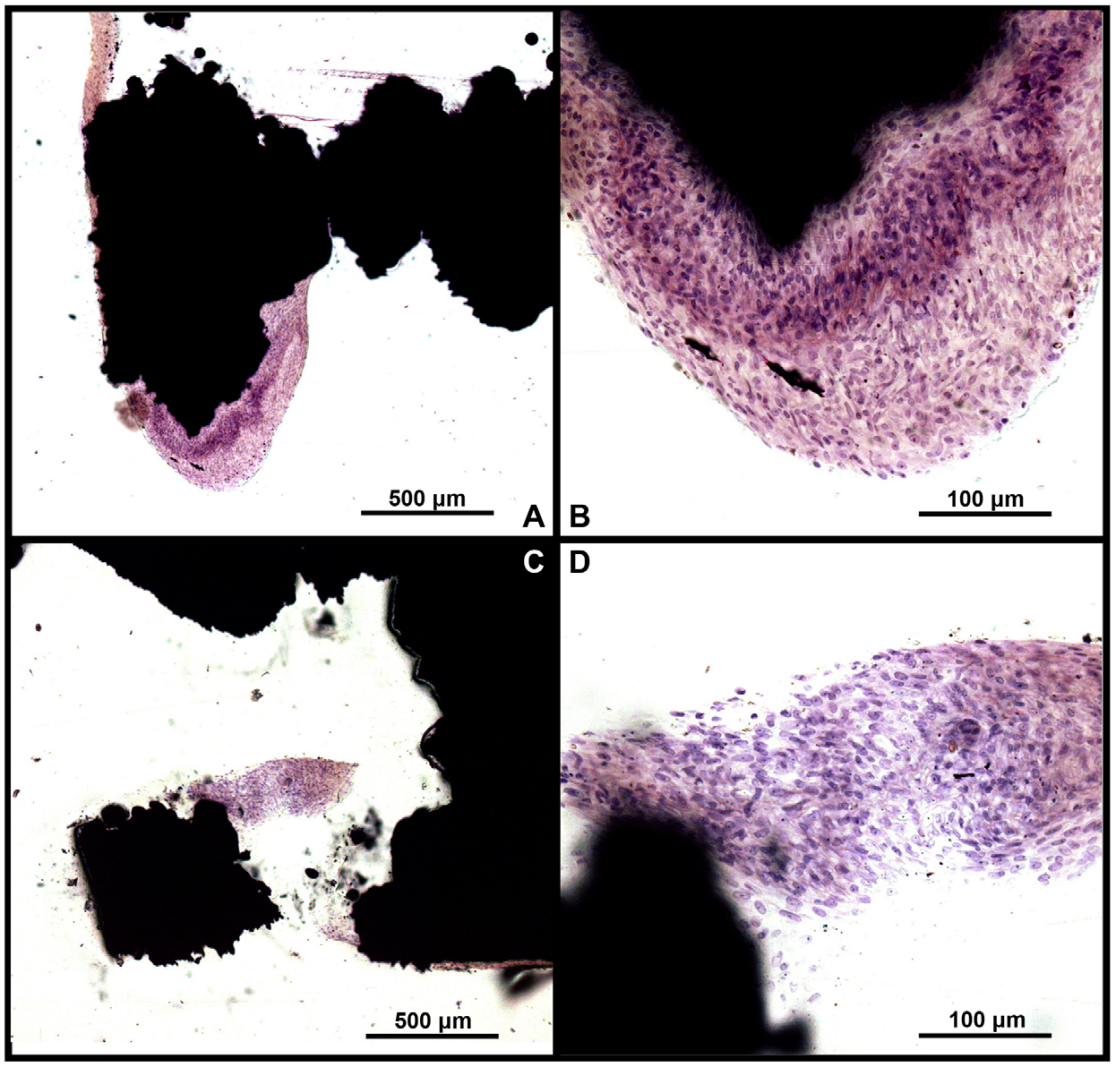
H&E stained sections of sample after 14 days of in vitro culture. Large amount of extracellular matrix deposited among the cells, and some cells migrated into the inner pore of both (**A–B**) uncoated and (**C–D**) coated samples.

**Figure 7:**
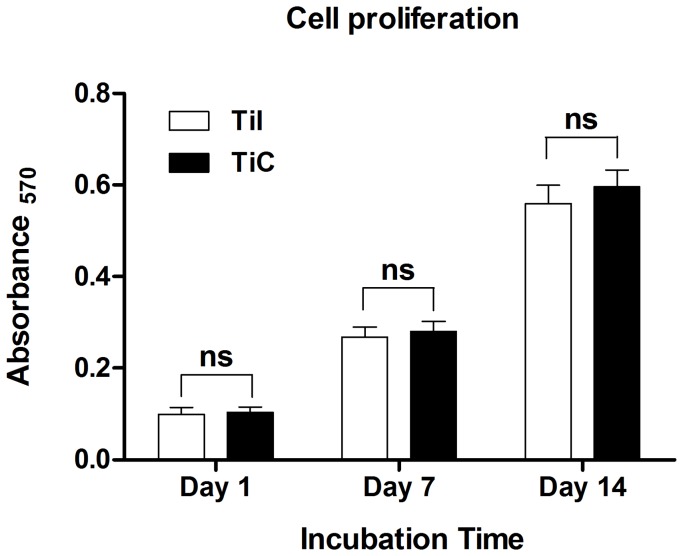
Cell viability of the sample over 14 days of in vitro culture. The cells on the porous titanium exerted a high and sustained proliferation rate within 14 days, and no significant difference was observed between the uncoated and coated samples at each timepoint (*P*>0.05). TiI, pure porous titanium implant; TiC, porous titanium implant with biomimetic coating.

### Characterization of Implant Surface with Biomimetic Coating

After immersion in SBF for 14 days, the specimens were rinsed in distilled water and dried in vacuum desiccator. Surface morphologies of the samples after chemical treatment and soaking in SBF were examined by scanning electron microscope. The thin film X-ray diffraction (TF-XRD) measurement was performed on a Rigaku X-ray diffractometer (Cu Kα radiation, 40 kV, 30 mA). The data was collected in the 2θ ranges of 20–60° with a step size of 0.02°. FTIR spectra were measured in transmission using the KBr technique in the range from 4000 to 400 cm^−1^ at a resolution of 4 cm^−1^. Approximately 1 mg of the coatings formed on the chemically treated titanium sample after soaking in SBF solutions for 14 days was removed from the substrate, then mixed with 500 mg of dry KBr powder and ground using an agate mortar and pestle. The resulting mixture was pressed into transparent pellets with diameter of 13 mm and applying force of 10^5^ N [27]. Both the EBM Ti6Al4V implants (TiI) and biomimetic apatite coated implants (TiC) were used for in vitro and in vivo analysis in the present study.

**Figure 8:**
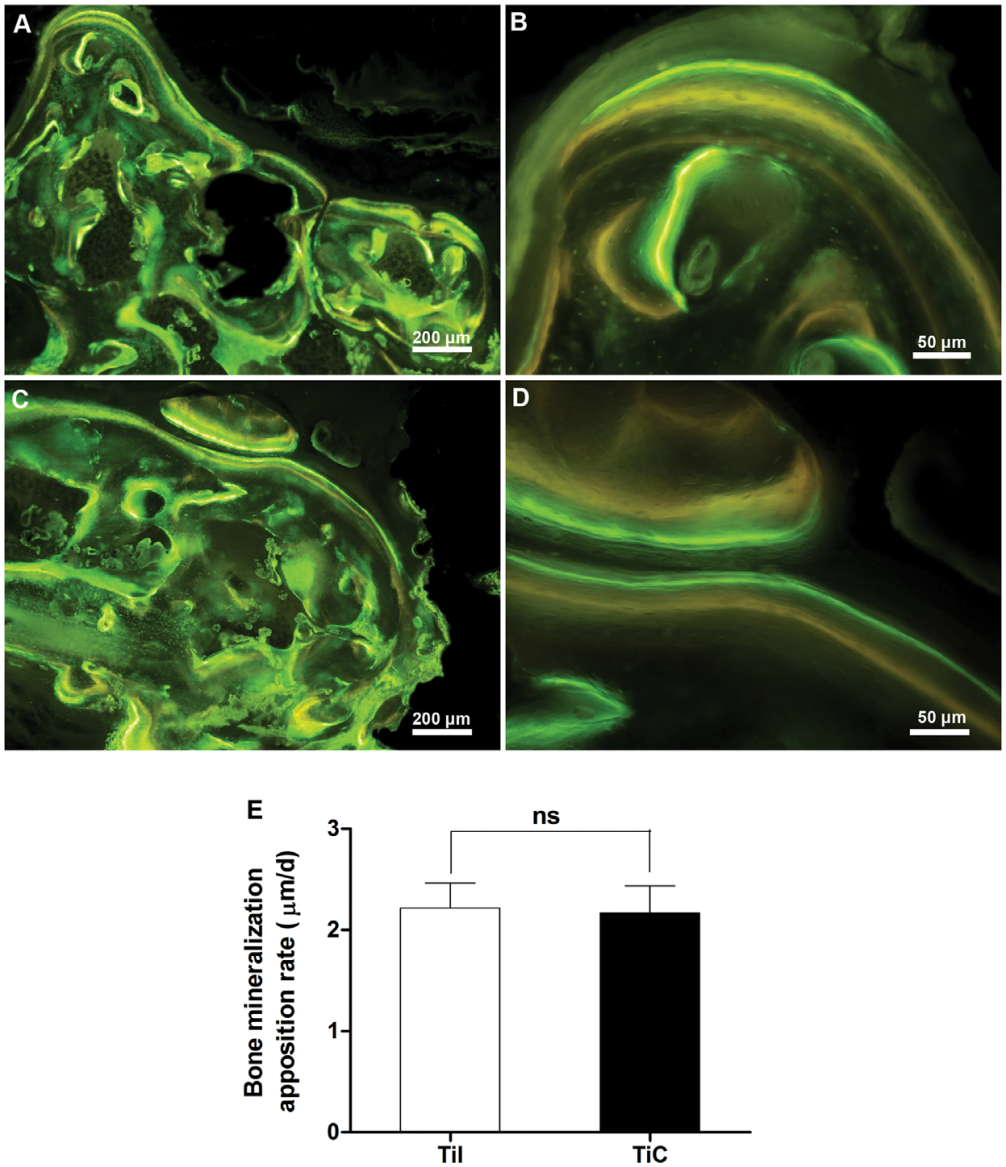
Fluorochrome labeling of bone regeneration at 12 weeks post-surgery. The fluorescent labeling indicated that abundant new bone growth into the porous titanium and continuous process of bone remodeling in both (**A–B**) uncoated and (**C–D**) coated samples. (**E**) The rate of bone mineralization apposition was similar in these two kinds of porous titanium at 12 weeks post-surgery (*P*>0.05). TiI, pure porous titanium implant; TiC, porous titanium implant with biomimetic coating.

### Cell Seeding and Co-culture with Implant

All samples were sterilized in ethylene oxide gas before cell seeding. Osteoblastic cells were isolated from new born New Zealand rabbit’s calvaria [28]. Cells were cultured in DMEM supplemented with 10% BCS and 1% penicillin/streptomycin and incubated at 37°C in a 5% CO_2_, 100% relative humidity incubator. The third passage cells were used in the present study. Osteoblasts were seeded on the scaffolds at a density of 6×10^5^ cells/ml.

**Figure 9:**
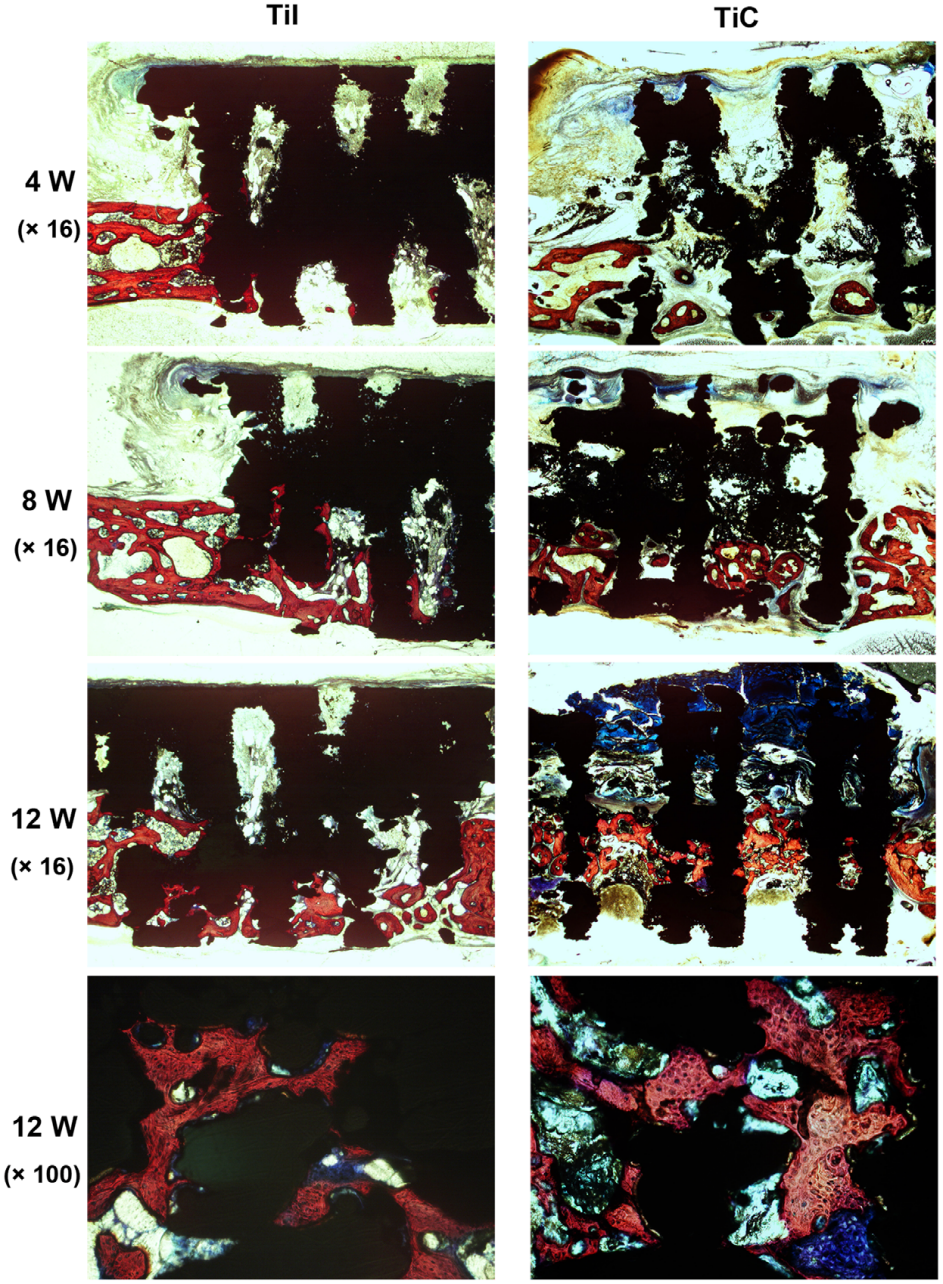
Histological staining for osteogenesis within porous titanium after implantation for 4, 8 and 12 weeks. The images showed that the rapid increase of bone ingrowth into the pores of titanium throughout the experiment and close contact between bone tissue and EBM implants at 12 weeks post-surgery, while there were no obvious differences between the uncoated and coated samples at each timepoint. TiI, pure porous titanium implant; TiC, porous titanium implant with biomimetic coating.

**Figure 10:**
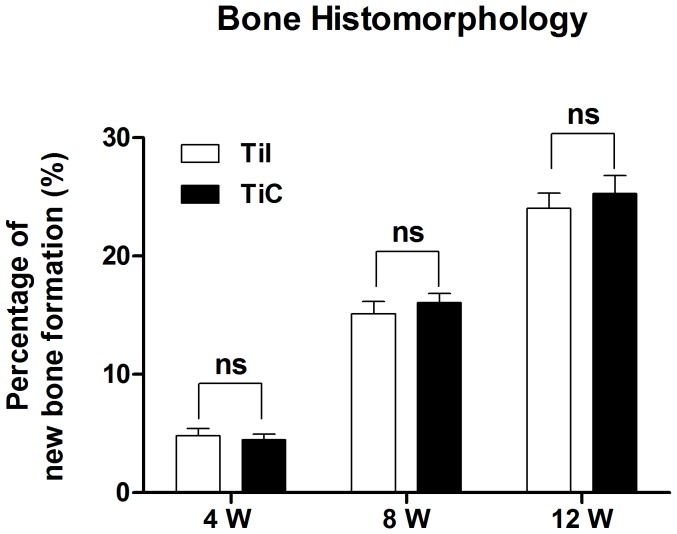
Histomorphometric analysis of new bone formation. The data showed a high percentage of newly formed bone in the defect region in both coated and uncoated implants during the whole experiment (*P*>0.05). TiI, pure porous titanium implant; TiC, porous titanium implant with biomimetic coating.

### Cell Attachment and Morphology

Samples were removed from culture and washed with phosphate buffered saline after 14 days incubation, and then fixed in 2% v/v glutaraldehyde in 0.1 M sodium cacodylate buffer at 4°C overnight for attachment and morphological observation. The samples were washed in the buffer, dehydrated through an ethanol series, critical-point dried, lastly sputtered with gold, and were analyzed using a scanning electron microscope (SEM) operating at 15 kV and a semiautomatic interactive image analyzer.

### Histological Examination in vitro

Samples for histological study were fixed in 10% formalin for 7 days, dehydrated through an ethanol series, cleared with toluene, and embedded in methylmethacrylate. After polymerization, thin sections were prepared with a modified sawing microtome technique. The sections were stained with Haematoxylin and Eosin (H&E) and examined with a standard microscope (Leica, Wetzlar, Germany).

### Cell Proliferation

Cell proliferation was assessed using a methylthiazol tetrazolium (MTT) assay. Briefly, 800 µl serum free medium and 80 µl MTT solution were added to each sample. After 4 h of incubation at 37°C in a fully humidified atmosphere with 5% CO_2_ in air, MTT was taken up by active cells and reduced in the mitochondria to insoluble purple formazan granules. Subsequently, the medium was discarded, and the precipitated formazan was then dissolved in dimethyl sulfoxide (DMSO). After 10 min of slow shaking, the absorbance was read at 570 nm using a Bio-Rad 500 spectrophotometric microplate reader.

### Implantation Assay

All animal experiments were performed in accordance with the National Institutes of Health Guidelines for the Use of Laboratory Animals. The animals used in the current study were obtained from protocols approved by the Fourth Military Medical University Committee on Animal Care (Permit Number: 12090). Thirty male New Zealand white rabbits, weighing 2.5–3.0 kg, were used to examine the implant-bone integration and bone ingrowth of the EBM Ti6Al4V porous implants of orthogonal structure with or without biomimetic apatite coating in experimental cranial implantation. All animals were anaesthetized by intramuscular injection of Ketamine. Two 12 mm full-thickness calvaria defects were drilled. The implants were placed alternately in the right or left defect. The breath and heart rate of rabbits were carefully monitored to ensure that they were under anesthetic and painless before recovering from anesthesia. Each rabbit was administered 400,000 U of penicillin intraoperatively and on the first postoperative day to prevent infection. The animals were anaesthetized and sacrificed by intra-cardiac overdose of sodium pentobarbital at 4, 8 and 12 weeks after implantation.

### Fluorochrome Labeling

Sequential fluorochrome markers were administered after implantation. Tetracycline (30 mg/kg, Sigma, USA) was administered 2 weeks and Calcein Green (8 mg/kg, Sigma, USA) was administered 3 days before the animals were sacrificed using intramuscular injection. After the animals were sacrificed at 12 weeks post-surgery, the implants were retrieved for fluorescence analysis.

### Histological and Histomorphometric Analysis

All retrieved specimens were fixed in 10% formalin for 7 days. After fixation, the samples were dehydrated in a graded series of ethanol and embedded in methylmethacrylate. After polymerization, thin sections were prepared with a modified sawing microtome technique and stained with 1.2% trinitrophenol and 1% acid fuchsin (Von-Gieson staining). The qualitative analysis of bone formation and fluorochrome markers were performed using a light/fluorescence microscope (Leica LA Microsystems, Bensheim, Germany). Image analysis was performed using the Image-Pro Plus software (version 6.0, Media Cybernetics, USA). The amount of newly formed bone inside the defect was evaluated and expressed as percentage of bone area in total available pore space [(bone area/(total implant area − total scaffold area)×100%]. In addition, the bone mineralization apposition rate (MAR, vertical spacing between two fluorochrome markers/injection interval) was analyzed by from the images of fluorochrome labeling, which generally indicated the new bone growth rate.

### Statistical Analysis

All the values in the experiment were expressed as the mean ± standard deviation, and were compared using one-way analysis of variance (ANOVA). Differences at *p*<0.05 were considered statistically significant.

## Results

### Structural Characterization

The Ti6Al4V samples produced by EBM process were shown in Fig. 2A. The pore architectures of samples were examined using micro-CT and SEM. The reconstructed 3D images from micro-CT measurements and micrographs from SEM examination were shown in Fig. 2B and Fig. 2C–E, respectively. All samples exhibited fully interconnected porous networks. This is one of the most important requirements for tissue ingrowth. Some molten particles could be found in the pores. The three-dimensional topographies of the implants surfaces, illustrated in Fig. 3, exhibited a rough anisotropic surface. The roughness *R_a_* values of the top and lateral surfaces were in the range of 5–10 and 15–21 µm. The sample lateral surfaces were rougher compared to the top surfaces due to adherent molten powder particles. For porous implants with different internal structure, the geometric characteristics and mechanical properties of the samples were shown in Table 1. The orthogonal structure implants, which exerted better mechanical properties than two other kinds of implants, were used for the in vitro and vivo analysis.

### Surface Modification

In Figure 4A, the SEM photographs showed a homogeneous surface layer formed on the surface of implants. EDX analysis revealed calcium, magnesium, sodium and phosphorus to be presented in the surface. The detected Ca/P molar ratio was 1.65. The TF-XRD pattern of the sample surfaces after alkali-heat treatment and subsequent immersion in SBF for 14 days was shown in Fig. 4B. The TF-XRD pattern exhibited broad diffraction lines. The position of these diffraction lines indicated that the deposits are formed of poor crystalline hydroxyapatite. The phase composition of the layer was analyzed in detail using FTIR (Fig. 4C). The spectra showed featureless phosphate and carbonate bands. Intense and broad bands were assigned to oxygen-hydrogen groups, and stretching and bonding were observed at 3435 and 1642 cm^1^ respectively. A broad band at about 1420–1450 cm^−1^ from the carbonate groups was incorporated in the apatite structure. These carbonate groups gave rise to the band at 875 cm^−1^. A broad band in the range of 960 to 1200 cm^−1^ from *v_3_* P-O antisymmetric stretching vibration, indicated the deviation of phosphate ions from the ideal tetrahedral structure. The bending modes of the O–P–O bonds in the phosphates were found in the spectral range from 560 to 602 cm^−1^. HPO_4_^2−^ groups were detected at 1108 cm^−1^ (*v_3_* mode). Therefore, the precipitate formed in SBF was characterized in a poorly crystallized or amorphous carbonated Ca-P phase.

### Cell Attachment and Morphology

After 14 days of culture, it was found that cells adhered to the modified surface, spread out and proliferated into the multilayer structures. The osteoblasts achieved confluence and cover the Ti surface completely with large amount of extracellular matrix (ECM) deposited among the cells, as demonstrated on the SEM images (Fig. 5) and H&E stained section (Fig. 6) of the cell/implant constructs. In addition, some cells have migrated into the inner of porous implants, but they did not span across the total implant. The cross-section histological staining showed a decrease of cell number from top to bottom of porous implants. Moreover, there was no obviously difference in morphology and dense of cells between porous implants with and without biomimetic coating.

### Cell Proliferation

MTT assay was used to study the cellular viability and proliferation of the cell culture in the biomimetically modified samples. It showed the maintenance of high cellular viability and a rapid proliferation of cells within 14 days (Fig. 7). The cell viability on EBM porous implant was similar to that on the biomimetic apatite-coated implants at each timepoint (all P>0.05).

### Fluorochrome Labeling

Bone remodeling was validated by interrupted fluorescent bone labeling and new bone deposition. The labeling was detected in the regenerated bone in the pores of EBM porous implants, revealing that the increase rate of bone deposition progressed into the pores of the scaffolds (Fig. 8A–D). The fluorescent labeling indicated similar bone ingrowth from the bone-implants interface to the center of the defect in pure porous implants compared with biomimetic apatite-coated porous implants, as well as the fluorescent labeling analysis showed that there were no differences in MAR between the coated (2.22±0.25 µm/d) and uncoated implants (2.18±0.27 µm/d, P>0.05) (Fig. 8E), indicating similar rate of bone ingrowth within the porous titanium with and without biomimetic apatite coating.

### Histological and Histomorphometric Analysis

To evaluate the tissue response to the implanted scaffolds and the defect healing progress, we performed histological analysis on the tissue/implants interface and the internal area of the implants. For all Ti6Al4V implants, no foreign body or inflammatory reaction was observed. Bone formation was found to start from the host bone bed towards the implant in all implants. Four weeks after surgery, histological observations revealed that a small amount of new immature bone tissue grew from the rim of bone defect and began to integrate with the periphery of porous implants, with the direct deposition of newly formed bone onto the surface of implants (Fig. 9A–B, bone tissue in red color while Ti6Al4V implant in black color). After 8 weeks of implantation, histological evaluation indicated progressive growth of more newly formed bone from the calvarial margins toward the center of the bone defect. The newly formed bone integrated well with implant surface (Fig. 9C–D). At 12 weeks, the newly formed bone has successfully bridged the calvarial defect, and the majority of the pores at the bottom part of the implants were filled with bone tissue, which were directly bridged with the calvarial defects (indicated in red color Fig. 9E–F). In addition, the process of bone remodeling was spotted in all the implant sites. Quantitative histomorphometric analysis demonstrated a rapid and continuous new bone formation in the defect region in both coated and uncoated implants at each timepoint during the whole experiment (all P>0.05) (Fig. 10).

## Discussion

The EBM process is a new additive manufacturing technique with great capability to fabricate dense and porous Ti6Al4V implants with better control on the both internal structure and external shape [29], [30], [31], [32]. In addition, the EBM production process is much faster comparing with conventional manufacturing methods using laser beam. In EBM process, the high power electron beam is used instead of conventional laser beam. With regards to energy utilization efficiency (EUE) during metal sintering, high power electron beam can be as high as 95 percent, which is 5 to 10 times higher than the EUE of the laser beam; whereas laser beam may lose up to 95% of its energy, due to the light reflection by the metal powder during the sintering. This leads to 3 to 5 times faster processing speed of EBM than other laser beam based metal additive fabrication methods. Furthermore, thanks to the high EUE of electron beam, EBM process provides a better and more reliable fabrication process compared to selective laser sintering (SLS), by fully melting the metal particles to manufacture high quality of implants, which is completely void-free. The whole EBM process occurs in an ultra high vacuum environment, eliminating any imperfections caused by oxidation.

The Young’s modulus of solid Ti6Al4V implants produced by Arcam EBM is up to 120 GPa [32]. In comparison with the solid ones, the Young’s modulus of porous Ti6Al4V implants in current study is similar to cortical bone, which could minimize stress shielding. It is obvious that the porous structures help to reduce the mechanical mismatches between metallic implants and host bone tissue. The ideal porous implants should provide a good mechanical environment for initial function and appropriate remodeling of regenerating tissue while concurrently providing sufficient porosity for cell migration and tissue ingrowth. Whereas, these requirements may lead to the conflicting design goals. Hollister et al. [33], [34] developed a general design optimization scheme for the pore architecture to match desired elastic properties and porosity simultaneously, by introducing the homogenization-based topology optimization algorithm. Furthermore, they demonstrated the prototypes of the designed structures which can be fabricated using additive manufacturing technique. Various porous structures cause different mechanical properties. Generally speaking, with increase of the porosity, the mechanical properties such as stiffness and compressive strength decreased [14]. It was found that not only porosity has an influence on the mechanical properties of porous samples, but also the pore size and orientation, and strut size of samples affect the mechanical properties [12], [14]. In current study, we pre-designed the porosity, pore size, and strut size of samples to adjust the mechanical properties to match that of cortical bone. The EBM-produced porous Ti6Al4V implants showed a favorable mechanical property (compressive strength range from 163–286 MPa, Young’s modulus range from 14.5–38.5 GPa) and suitable porous structure (fully interconnected porous networks, porosity range from 51–61%, the pore size range from 500 - 600 µm) for the load bearing application in treating large segmental bone defects.

The biomimetic technique allowed the homogeneous deposition of a carbonated apatite layer onto the porous metallic implants [14], [25]. This coating has a composition and crystallinity index similar to that of bone mineral [23]. Jalota et al. investigated the proliferation of osteoblast on neat and bone-like apatite coated surfaces of titanium foams and found that bone-like apatite coated surfaces exhibited the highest protein production and cell attachment [35]. It was revealed that the direct bone contact was significantly higher for bone-like apatite coated dense Ti6Al4V than the corresponding uncoated implants [25].

As one part of our study design, the biomimetic approach was used to deposit bone-like apatite on porous samples surfaces to promote the attachment, migration and proliferation of osteoblasts on the samples. However, no significant differences were observed in osteoblast function and morphology on the porous scaffolds between groups with or without biomimetic coatings. Ponader et al. reported that topographical surface modifications of electron beam melted Ti-6Al-4V titanium markedly influenced the function of human fetal osteoblasts [36]. The interactions between cells and microtopography of scaffold have been extensively studied, suggesting that microtopographies can promote bone-to-implant contact via such mechanisms as mechanical interlocking [37] and enhancement of osteoblast functions [38]. Cells can well communicate and in consequence proliferate as long as there is enough space to attach on the relatively smooth surface [39], while the peaks on the rough surfaces of implants may offer more favorable biological environment for the adhesion and differentiation of osteoblasts [40], [41]. The EBM titanium in the present study consist of Ti6Al4V powders (particle size: 45–100 µm) [12] which provide mild undulant surfaces without high peaks and scarped flanks, making it suitable matrix to trigger the attachment and proliferation of osteoblasts.

The in vivo experiment revealed that bone had started to grow from the host bone bed to the porous implants. The penetration depth of new bone regeneration increased rapidly with the implantation time, and eventually bridged the defect at week 12, demonstrating excellent osteoconductivity of the Ti6Al4V implants fabricated by EMB process. The amount of formed bone of EBM Ti-6Al-4V titanium in our study was much higher than that of porous titanium manufactured by other methods [29], and was comparable to that of EBM porous titanium reported by other study [31]. Takemoto et al. reported that the bioactive porous titanium achieved bone ingrowth to a depth of 3 mm within 4 weeks of implantation and continued to increase throughout the 16 weeks of implantation, whereas bone ingrowth into the nontreated implants tended to decrease between 4 and 8 weeks [42], whereas the pure EBM porous titanium used in our study exerted remarkable and continuous increase of bone formation throughout the whole experiment. Although several studies suggested that it might be necessary to improve the biological performance of titanium implant by being treated chemically and thermally, and/or coated with bioactive materials [43], [44], [45], it was similar for the new bone ingrowth within the implants with and without apatite coating. It was revealed that the properties that were considered as the important requirements for bone ingrowth contained favorable topographical surface for cell adhesion and proliferation, interconnected porous structures in all dimensions, appropriate mechanical strength corresponding to those of native bone [14], [31], [36]. In this study, it looks like the adequate architecture and mechanical properties of EBM porous scaffolds may contribute mainly to the optimized ingrowth of surrounding bone tissue in addition to the surface modification.

### Conclusion

EBM technique can be used to fabricate Ti6Al4V implants with well controlled porous structures and favorable mechanical properties, which is close to native bone tissue. This Ti6Al4V implants presented good cell attachment and proliferation properties in vitro, as well as satisfied bone ingrowth and direct bonding between host bone tissue and the implants. Biomimetic approach can be utilized to coat the porous Ti6Al4V implants with a homogenous layer of bone-like apatite, whereas this biomimetic coating did not further enhance the bioactivities of the titanium scaffolds. Therefore, porous Ti6Al4V implant fabricated by EBM technique possesses great potential for the clinical applications, which can not only reduce the mechanical mismatch but also achieve stable long-term fixation by promoting bone ingrowth. This study opens up the possibility of using high strength porous scaffolds with appropriate osteoconductive and osteogenic properties to reconstruct bone defects on specific sites in the maxillofacial and orthopedic fields.
